# Distribution of a Foliage Disease Fungus Within Canopies of Mature Douglas-Fir in Western Oregon

**DOI:** 10.3389/ffgc.2022.743039

**Published:** 2022-02-11

**Authors:** Yung-Hsiang Lan, David C. Shaw, E. Henry Lee, Peter A. Beedlow

**Affiliations:** 1 Department of Forest Engineering, Resources, and Management, Oregon State University, Corvallis, OR, United States; 2 U.S. Environmental Protection Agency, Pacific Ecological Systems Division, Corvallis, OR, United States

**Keywords:** Douglas-fir, fungal disease, foliar pathogen, *Nothophaeocryptopus gaeumannii*, Swiss needle cast, tree canopy

## Abstract

*Nothophaeocryptopus gaeumannii* is a common native, endophytic fungus of Douglas-fir foliage, which causes Swiss needle cast, an important foliage disease that is considered a threat to Douglas-fir plantations in Oregon. Disease expression is influenced by fungal fruiting bodies (pseudothecia), which plug the stomata and inhibit gas exchange. Trees are impacted when pseudothecia plug stomates on 1-year-old and older needles resulting in early needle abscission. Mature (100 years+) trees appear to be less impacted from disease, and we hypothesize this is due to the greater emergence of pseudothecia on older than younger needles, which allows for more needle retention. We measured the density of pseudothecia occluding stomates across 2- to 5-year-old needles from upper, middle, and lower canopy positions of mature trees at three sites in the Oregon Coast Range and two sites in the western Oregon Cascade Mountains. Binomial generalized linear mixed model (GLMM) was used to test for the effects of canopy position (upper, middle, and lower), sites, needle age (2–5 years old), and years (2016 and 2017), and their interactions on the pseudothecia density. Pseudothecia density varied annually depending on sites, needle age and canopy positions. Pseudothecia density peaked on 3-, and 4-year-old needles, however, needles emerging from the same year, like 2-year-old needles in 2016 and 3-year-old needles in 2017 both emerged in 2014, had consistently similar patterns of pseudothecia density for both years, across site and canopy positions. Canopy position was important for 3-, and 4-year-old needles, showing less pseudothecia in the lower canopy. This research confirms that *N. gaeumannii* pseudothecia density is greatest in 3- and 4-year old needles in mature trees in contrast to plantations where pseudothecia density usually peaks on 2-year-old needles, and that pseudothecia density (disease severity) is generally lower in mature trees. Something about mature forest canopies and foliage appears to increase the time it takes for pseudothecia to emerge from the needles, in contrast to younger plantations, thus allowing the mature trees to have greater needle retention.

## INTRODUCTION

**FIGURE 1 |:**
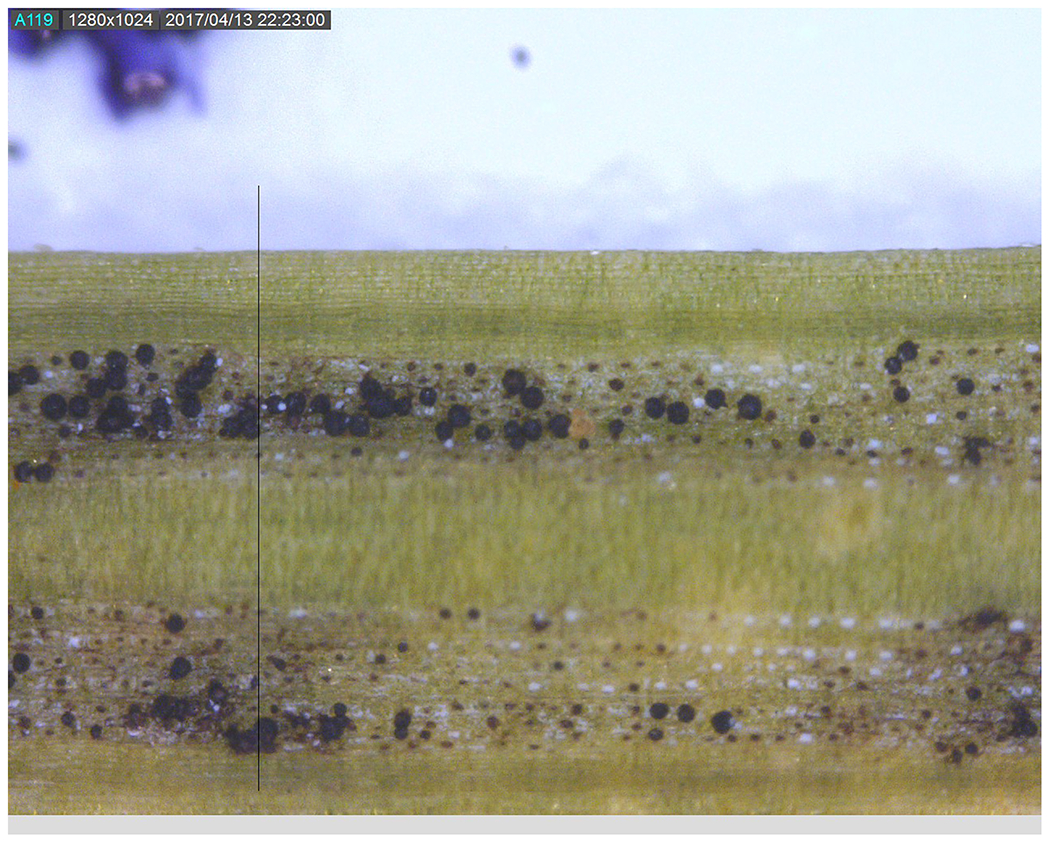
Photo of pseudothecia blocking stomates on the underside of a Douglas-fir needle.

*Nothophaeocryptopus gaeumannii* is a common native, endophytic fungus which occurs only in Douglas-fir (*Pseudotsuga menziesii*) foliage (Hansen et al., 2000). The fungus can cause a foliage disease known as Swiss needle cast, which is currently defoliating and decreasing growth of Douglas-fir along the Pacific Coast in Oregon, Washington, and SW British Columbia (Ritóková et al., 2016; Shaw et al., 2021). Disease is caused when the fruiting bodies of the fungus, known as pseudothecia (Figure 1), emerge from and plug the stomates which causes reduced gas exchange and carbon starvation (Manter et al., 2000). This fungus may be unusual for a pathogen in that newly emerged needles are the predominant substrate for new infection by ascospores (Rohde, 1937; Chastagner and Byther, 1983) and colonization within needles is exclusively intercellular and non-lethal to cells (Stone et al., 2008a). Swiss needle cast induced reductions in tree growth of coastal Douglas-fir stands vary in space and time depending upon elevation, aspect, proximity to the coast, and site conditions and primary environmental factors influencing the degree of pathogen dynamics (Rosso and Hansen, 2003; Coop and Stone, 2007). Evidence from dendrochronological studies indicate that Swiss needle cast impacts in Oregon were least severe in the first half of the 20th century and increased in frequency, severity and range after ~1980 due to increasing winter temperature associated with climate change (Black et al., 2010; Lee et al., 2013, 2017). The Swiss needle cast epidemic in the most recent decades is a primary economic and ecological concern to the timber industry in the Pacific Northwest because disease severity is greater in young coastal Douglas-fir plantations than in mature stands but reasons for this remains elusive (Lan et al., 2019; Mildrexler et al., 2019). Coastal young Douglas-fir trees typically retain up to 4 years of needles but may have only current and 1-year-old foliage in severely infected plantations (Hansen et al., 2000; Maguire et al., 2002; Zhao et al., 2011) due to early needle abscission when between 25 and 50% of the stomates are occluded (Hansen et al., 2000; Manter et al., 2003; Stone et al., 2008a). Anecdotal evidence from epidemiological studies suggest needle retention appears to drive the growth impacts on the tree, with foliage retention of only 2 years causing a reduction in growth of about 30% (Maguire et al., 2002, 2011; Shaw et al., 2021). Needle retention and fungal fruiting body presence and abundance on 1- and 2-year-old foliage have routinely been used as indices of disease severity in Douglas-fir plantations (Hood, 1982; Michaels and Chastagner, 1984; Hansen et al., 2000). However, disease severity on 2-year-old needles may be misleading for mature Douglas-fir trees which typically have greater needle retention of more than 5 years (Lan et al., 2019) and lesser frequency and magnitude of growth losses (Lee et al., 2017).

Recent epidemiological evidence suggests that disease severity, as measured by incidence times the percentage of pseudothecia occluding stomates (Mulvey et al., 2013), is greatest in 2-year-old needles for young trees, and 3–5 year-old needles for older trees (Lan et al., 2019). The reason for this difference remains elusive, as foliar nitrogen and leaf wetness did not explain the difference. The reason mature trees have greater foliage retention, and are healthier, may be influenced by the timing of pseudothecia emergence. While there is considerable epidemiological evidence of disease severity for young Douglas-fir plantations in coastal Oregon and Washington (Ritóková et al., 2016, 2021), the data and knowledge gaps of disease severity for mature Douglas-fir are large due primarily to comparably few epidemiological studies on mature Douglas-fir and scaling issues from sampling only 2-year-old needles for measuring incidence.

This manuscript extends our previous epidemiological study (Lan et al., 2019) to examine the differences in disease severity between needle age classes and canopy position of mature Douglas-fir trees based on within-leaf measurements of the density of pseudothecia occluding stomates. There is no published data on pseudothecia density of multiple needle age classes in mature trees. Lan et al. (2019) found a greater percentage of 3–5 year-old needles of mature Douglas-fir were infected by the presence of pseudothecia than younger needles, but the amount of stomata occluded by pseudothecia on infected needles (i.e., pseudothecia density) was not measured. Consequently, the pathogenicity of *N. gaeumannii* and epidemiology of mature trees are still not very clear. Because the physiological effects of SNC (impaired CO_2_ uptake and photosynthesis) are associated with the physical blockage of stomata, the density of pseudothecia occluding stomates is a relevant response variable for assessing disease severity across needle age classes and canopy positions of mature trees. We hypothesized that disease severity is less in mature trees because the time since initial infection to the emergence of pseudothecia is longer for mature than young trees. Within the mature tree crowns, we also explored whether pseudothecia density varied with needle age, canopy position, and site. We believe that this analysis provides important insight into spatial and temporal dynamics of Swiss needle cast disease progression in mature tree canopies. Also, filling a data gap of the differences in mature and young trees can lead to a better understanding of the ecology and epidemiology of this important foliage disease.

## MATERIALS AND METHODS

### Study Sites

**FIGURE 2 |:**
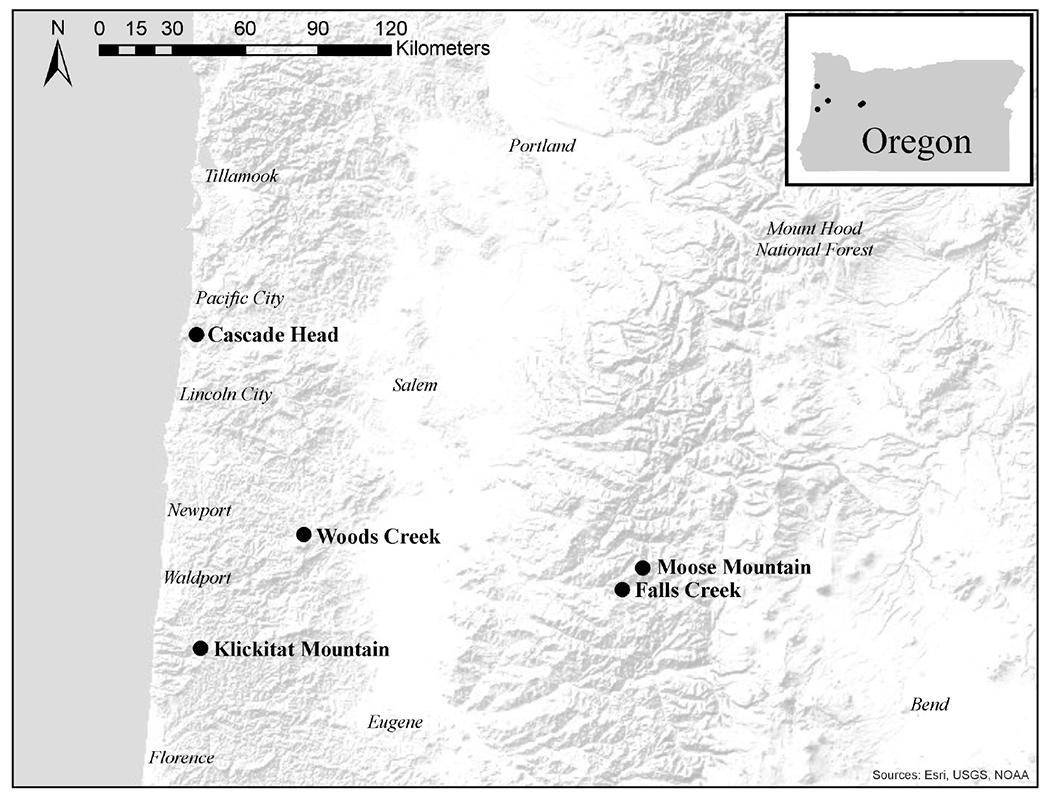
Map of study sites in the Oregon Coast Range and western Cascade Mountains of western Oregon, United States. Cascade Head, Falls Creek, and Moose Mountain are in the long-term ecological monitoring plot (LTEM) system, and Klickitat Mountain and Woods Creek are not part of the LTEM system. Cascade Head, Klickitat Mountain, and Woods Creek are in the Siuslaw National Forest. Falls Creek and Moose Mountain are in the Willamette National Forest.

**FIGURE 3 |:**
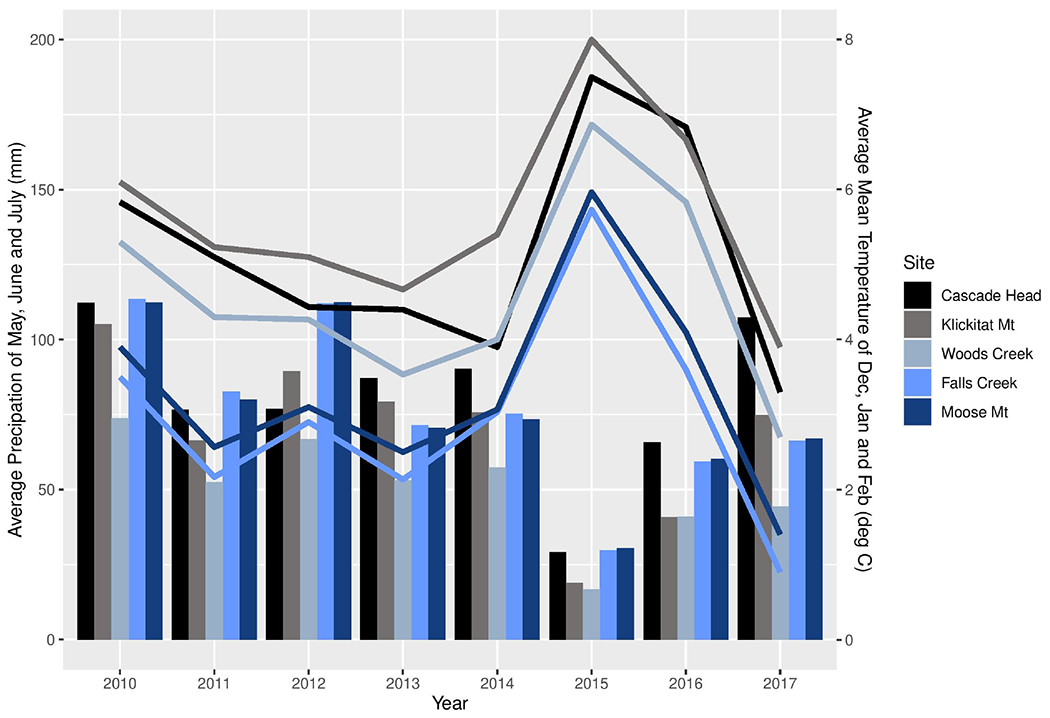
Average winter (December, January, and February) mean temperature (lines) and average summer (May, June, and July) precipitation (bars) of each study site from 2010 to 2017.

We sampled the same mature trees from five of the seven sites in western Oregon that were included in the study by Lan et al. (2019); three sites were in the long-term ecological monitoring plot (LTEM) system established by the United States Environmental Protection Agency (hereafter EPA; Lee et al., 2007; Beedlow et al., 2013) and two sites were not part of the LTEM system. Moose Mountain and Falls Creek are located on the west slope of the Cascade Range in the Willamette National Forest, and Cascade Head, Woods Creek, and Klickitat Mountain are in the Siuslaw National Forest in the Coast Range (Figure 2). The forests were unmanaged mature stands of Douglas-fir that were 120–150 years old and ranged in height from 55 to 70 m. Elevation of sites varied from 140 to 670 m. Annual precipitation varied from 1700–2500 mm. Associated tree species included western hemlock (*Tsuga heterophylla*) and western red cedar (*Thuja plicata*), in Cascade Head Sitka spruce (*Picea sitchensis*) is also associated. Monthly precipitation and mean temperature data from 2010 to 2017 at all sites were downloaded from PRISM at Oregon State University^1^ by providing GPS coordinates. We use average winter mean temperature (December, January, and February) and average summer precipitation (May, June and July), the climatological variables that are associated with Swiss needle cast severity (Manter et al., 2005), to compare the weather conditions across the sites (Figure 3).

### Field Sampling

Branch samples were collected on the south side of each tree in late May through early June in 2016 and 2017, after bud-break and before new branchlets were elongated. The May–June period is also optimal for sampling because *N. gaeumannii* asci mature during April–June in western Oregon and sporulation occurs mid-May to July (Rohde, 1937; Chastagner and Byther, 1983; Michaels and Chastagner, 1984). We collected 1–3 branches from three canopy positions (lower, middle, and upper crown) in each of three trees at each site (total number of trees = 15). At least one branch > 1 m in length was selected to ensure sufficient needle material for measurements. Several shorter branches were chosen if there were no branches greater than 1 m in length. Branches were transported to the lab and stored in a 5°C cold room. Three young trees (20 – 30 years old) next to the mature trees at each site were sampled at the same time by Lan et al. (2019) and data are used here for comparison to older stands.

### Lab Analysis

For each canopy position of 15 mature trees, 50 individual needles were randomly selected from the cohort of each foliar age class and needles were taped on an index card and stored at −20°C. All needle age classes from 2 to 6 years old were examined for *N. gaeumannii* pseudothecia incidence and density. The pseudothecia incidence is defined as the percentage of the 50 needles with pseudothecia present. Pseudothecia density was determined by selecting the first 10 needles with pseudothecia present beginning at the top of the card and working down, and then counting the percent of stomates occluded in three regions (base, middle, and tip) of the needle (Mulvey et al., 2013). The entire length of the needle was evenly divided into three regions, for each region we picked a starting point using a random number table, and examined 100 contiguous stomates from the starting point to determine the number that were occluded by pseudothecia. Pseudothecial occlusion in the three regions was then averaged for each needle and then averaged for 10 needles per canopy position per tree.

Disease severity is typically determined by multiplying the incidence times the density of stomates blocked by pseudothecia for 2-year-old needles (Mulvey et al., 2013; Ritóková et al., 2021; Shaw et al., 2021). Disease severity is an important metric used to compare disease importance in forest stands (Shaw et al., 2021), while density alone is most important in casting of individual needles (Hansen et al., 2000; Manter et al., 2003; Stone et al., 2008a). The 2-year-old foliage is the key age class in coastal forests but foliage retention of severely diseased trees can be less than 2 years (Ritóková et al., 2016). Where disease severity is lower, pseudothecia can take as long as 4 years to mature when environmental conditions are less favorable for fungal growth (Stone et al., 2008a). In this study, we measured density of stomates plugged with pseudothecia for needles aged 2–5 years and included incidence data from Lan et al. (2019). We used the pseudothecia occlusion density rather than incidence as the primary measure of disease expression because there were many incidence values at or near zero.

### Statistical Analysis

Binomial generalized linear mixed model (GLMM) was used to test for effects of canopy positions (upper, middle, and lower), sites, needle ages (2–5 years old), years (2016 and 2017), and their interactions on the pseudothecia density at the 0.05 level of significance. In the GLMM, canopy position, site, and needle age were treated as fixed effects with interaction terms in the GLMM whereas year was treated as an additive fixed effect, the individual trees on the same site were treated as random effects. We first ran the GLMM by using only 2-year-old needles of mature and young trees, to confirm the statistical differences of pseudothecia incidence and density between mature and young trees. We analyzed pseudothecia incidence and density ratios (0–1) using binomial GLMM assuming a logit-linear model with four fixed factors and one random factor for individual trees.

To understand more deeply about pseudothecia density on mature trees, we first ran the GLMM by using the complete dataset. In the preliminary results, there were interactions involving canopy positions, sites, and needle age, so we also ran the GLMM by year and by needle age, to test for differences in pseudothecia density on canopy position and site separately. We conducted a Bonferroni mean separation test to infer which treatment means were different. GLMM tests were performed using R (v. 4.0.2, R Core Team, 2020) and package dplyr (Wickham et al., 2017), emmeans (Lenth, 2018), ggplot2 (Wickham, 2009), nlme (Pinheiro et al., 2017), glmmTMB (Brooks et al., 2017), and multcomp (Hothorn et al., 2008).

## RESULTS

Mature Douglas-fir trees were less severely infected by *N. gaeumannii* than nearby young Douglas-fir trees (10–20 years old) based on examination of pseudothecia incidence and density for 2-year-old needles (Table 1). The mean percentage of 2-year-old needles with pseudothecia present, or incidence, ranged from 1.3 to 98% across sites and years for mature Douglas-fir trees, compared with 44 to 100% for the young plantations (Table 2). Tree age differences (i.e., mature vs. young trees) in pseudothecia incidence and density for 2-year-old needles were highly significant at the 0.05 level of significance (*p* = 0.02 and *p* < 0.001 respectively, Table 1). To a lesser extent, site and canopy position effects and their interactions on incidence and density for 2-year-old needles were also significant at the 0.05 level of significance based on the binomial GLMM, however, year effect was significant only in pseudothecia incidence but not density (Table 1).

Pseudothecia incidence was uniformly high for all needle classes of mature trees at Cascade Head where disease severity was greatest. For less severely diseased stands of mature trees, incidence varied by needle age class and peaked for 3- and 4-year old needles rather than for 2-year-old needles. The mean percentage of occluded stomates on 2-year-old needles with pseudothecia present, or pseudothecia density, ranged from 0.1 to 6.5% for mature trees in comparison to a range of 2–21% for young plantations. The highest pseudothecia density recorded for mature trees were: Cascade Head 9.3% for 6-year-old needles, Klickitat Mountain 4.0% for 5-year, Woods Creek 8.4% for 4-year, Moose Mountain 8.8% for 4-year, and Falls Creek 8.6% for 3-year old needles (Table 2). Because of the limited sample size in this study, the variation of pseudothecia density and incidence between tree to tree could be large (Table 2).

**FIGURE 4 |:**
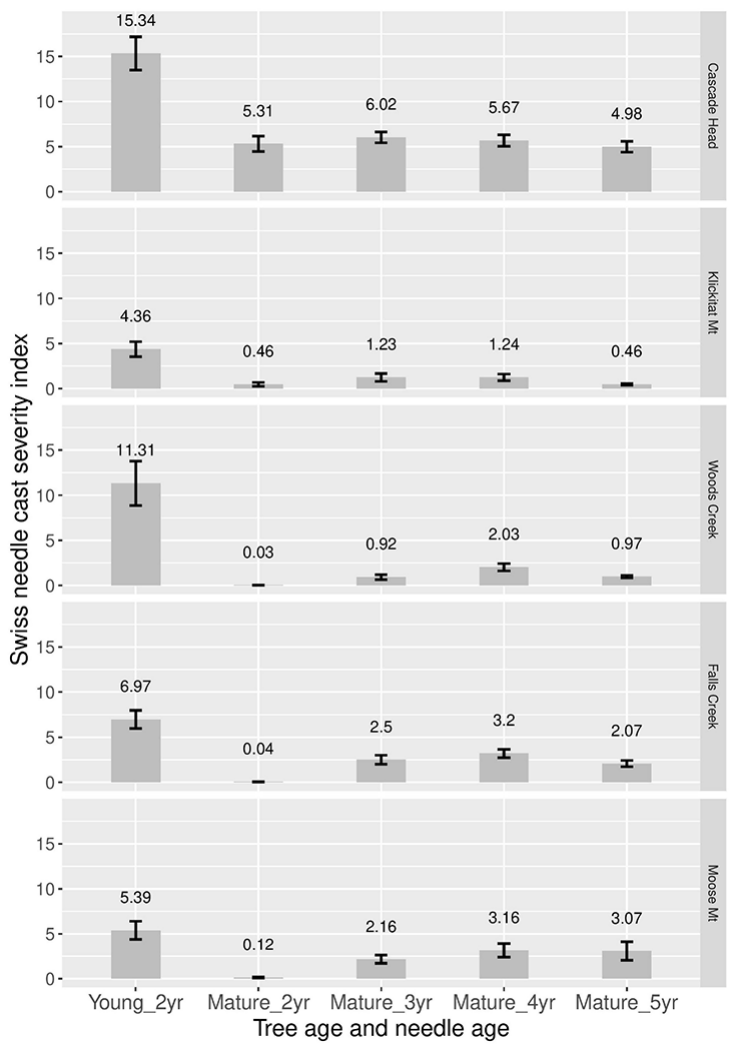
Swiss needle cast average disease severity (incidence × density) for 2-year-old needles in young nearby plantations, and for 2, 3, 4, and 5-year old needles in mature stands. Two years and three canopy positions were averaged at each site.

Disease severity (incidence times density) was consistently greater for 2-year-old needles of young plantation trees than for 2–5 years old needles of mature trees (Figure 4). Disease severity was greatest for 3- and 4-year old needles of mature trees, although 5-year-old needles were similar to 4-year old needles at Cascade Head and Moose Mountain.

### Within Crown and Site Patterns of Mature Trees

**FIGURE 5 |:**
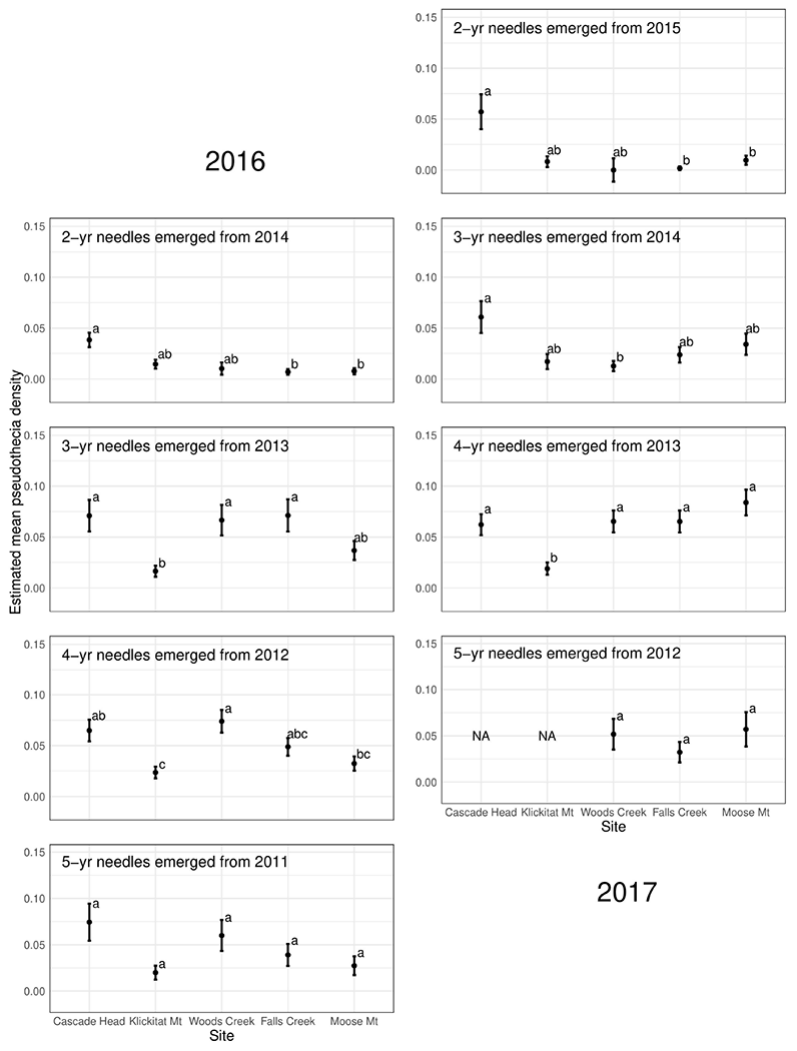
Pseudothecia density by site for 2- to 5-year old needles in 2016 and 2017 from mature Douglas-fir trees in western Oregon. Sites listed on the *X*-axis are from coast (left) to inland (right). Different letters represent that the two groups are statistically different.

**FIGURE 6 |:**
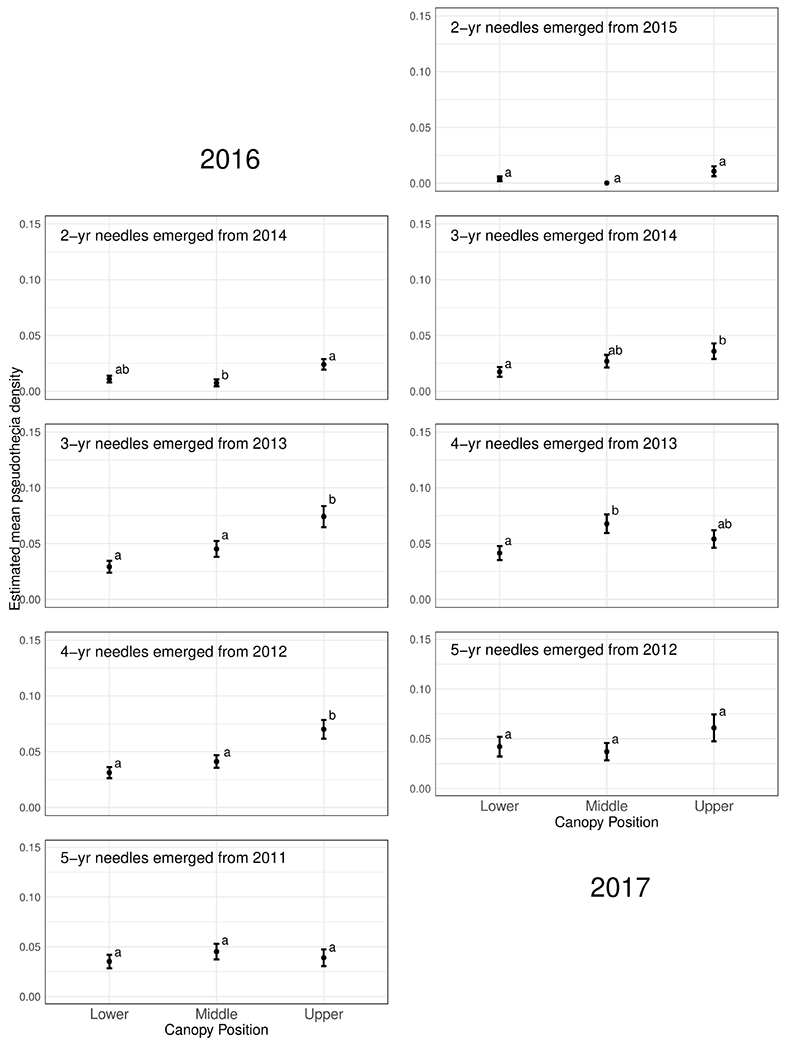
Pseudothecia density by canopy position for 2- to 5-year old needles in 2016 and 2017 from mature Douglas-fir trees in western Oregon. Different letters represent that the two groups are statistically different.

Pseudothecia density varied by canopy position, site, and year depending upon needle age class (Figures 5, 6 and Table 3). Site differences in pseudothecia density of 2-year-old needles were statistically significant at the 0.05 level, with greater pseudothecia density at the Cascade Head site than the inland sites (Figures 4, 5). Canopy position differences in pseudothecia density of 3- and 4-year-old needles were statistically significant at the 0.05 level, with greater pseudothecia density in the upper canopy than in the lower canopy (Figure 6).

Site differences in pseudothecia density were statistically significant at the 0.05 level of significance (Table 3), indicating that temporal and spatial variability might exist in response to local weather variability. Only current year foliage is infected by *N. gaeumannii*, and variability of weather during the year foliage emerges can influence infection success (Stone et al., 2008a). Year-to-year variation in temperature in winter and rainfall in summer is common (Figure 3). Pseudothecia density of 2-year-old needles was greatest at Cascade Head, notably in 2017, whereas that of 4-year-old needles in 2016 was greatest at Woods Creek (Figure 5 and Table 2). Pseudothecia density peaked in either 2- or 3-year-old needles at Cascade Head and 3- or 4-year-old needles at the inland sites depending upon year (Figure 5).

Canopy position differences were statistically significant at the 0.05 level (Table 3). Needles from upper canopy position had significantly higher pseudothecia density than the needles from middle (*p* = 0.024, Table 3) and lower canopies (*p* < 0.001, Table 3). In addition, these trends in canopy position also persisted over time for needles that emerged from the same year. For example, the canopy position trends in 2-year-old needles in 2016 was similar to the canopy position trends in 3-year-old needles in 2017, which they both emerged from 2014. The canopy position trends are particular for the 2- and 3-year old needles in 2016. In contrast, the canopy position trends in 3-year-old needles in 2016 did not persist in 4-year-old needles in 2017 due, in part, to a combination of pseudothecia density in the upper canopy peaking in 2016 followed by greater needle abscission in 2017 (Figure 6). The needle cohorts that emerged from the same year had similar trends among sites, implying a weather effect.

## DISCUSSION

Swiss needle cast is an important disease of plantation Douglas-fir along the coast, yet mature stands are apparently not currently severely infected except in limited areas such as Tillamook, Oregon (Black et al., 2010). Older trees have lower disease severity on 2-year-old needles (Lan et al., 2019) and older stands rarely show up in Swiss needle cast aerial detection surveys (Mildrexler et al., 2019). In this study, pseudothecia density, as well as disease severity, peaks on 3-, and 4-year-old needle age classes in mature Douglas-fir trees (Figure 4). This is consistent with previous findings that pseudothecia incidence peaks on 3-year or older needles for mature Douglas-fir (Lan et al., 2019). Pseudothecia density is also very low across all our mature tree samples compared to young tree 2-year-old needles, with density never exceeding 10% for any needle age class (Table 2). Manter et al. (2000) states that carbon assimilation decreases proportionally with increasing stomate occlusion, while Hansen et al. (2000) suggest leaves are cast above 50% occlusion and Manter et al. (2003) suggest casting with as low as 25% stomate occlusion. Therefore, it is likely that the levels of stomate occlusion found in older trees do not influence needle function as strongly as in younger trees, although we did not test this.

The combination of lower pseudothecia density and the emergence of pseudothecia on 3- or 4-year-old needles, rather than 2-year-old needle age class, is potentially the key difference between Swiss needle cast disease expression in young and mature Douglas-fir. This supports our hypothesis, that the reason mature stands do not typically suffer from disease caused by *N. gaeumannii* is because needle retention is typically above 3 years (Lan et al., 2019). The lower pseudothecia density on mature trees also makes needle casting due to disease less likely in mature needles.

### Why Do Pseudothecia Emerge on Older Needles in Mature Forests?

The factors that control timing of pseudothecia emergence from needles are not well understood, yet they may be critical to explaining disease epidemiology because the *N. gaeumannii* is an endophyte that appears to only impact leaves when pseudothecia emerge and block gas exchange (Manter et al., 2000, 2005). Manter et al. (2005) has shown that winter temperature may explain timing of pseudothecial development, while Lan et al. (2019) showed that leaf wetness and leaf nitrogen do not explain differences in Swiss needle cast disease severity using 2-year-old needles of young and mature trees. Evidence from dendrochronological studies suggest winter temperature is strongly associated with disease impact at wetter, cooler sites while summer conditions are more important at less humid, warmer sites (Lee et al., 2013). In 2015, the PNW experienced the warmest winter on record with winter temperatures 3.4°C above historical average (Mote et al., 2019). The anomalously warm winter of 2015 likely contributed to the high disease severity at the coast site while below-average spring and summer precipitation and above-average summer temperatures in 2015 likely decreased pseudothecia incidence and density at inland sites outside of the coastal fog zone (Lee et al., 2013, 2017).

### What Factors Could Control Timing of Pseudothecia Emergence?

Although we did not specifically test why pseudothecia emerge on older needles of mature trees, canopy architecture is distinct between young plantations and mature tree crowns. The mature trees are taller, with longer vertical foliage distribution and more complex microclimatic variation (Parker et al., 2002). Leaf temperature in winter may be reduced in mature trees compared to younger stands due to age differences in canopy height and architecture, and this could slow the development of pseudothecia production (Manter et al., 2005). Spore dispersal could also be different within older forest stands with complex crowns compared to even-structured young stands where canopy connectivity and homogeneity may allow for better colonization of needles. Other factors that might influence pseudothecia development time could be related to differences in leaf structure or chemistry between young and mature trees (Lan et al., 2019).

### Patterns Within Mature Tree Crowns

We found that *N. gaeumannii* pseudothecia density varies year to year depending on needle age and canopy position in mature Douglas-fir of western Oregon. Needles emerging from the same year, for example, 2-year-old needles in 2016 and 3-year-old needles in 2017 both emerged in 2014, have similar patterns of pseudothecia density across needle ages which likely relates to differences in weather during the year that needles are infected (Figure 5). *Nothophaeocryptopus gaeumannii* only infects the current-year needles from May–August when pseudothecia disperse spores (Michaels and Chastagner, 1984) and leaf wetness is important for fungal colonization (Manter et al., 2005). The weather during these months may cause the similar pseudothecia occlusion for the same-year needle cohorts due to similarities in weather-driven fungal colonization. The site effect was important for 2-year-old needles in our study, with highest pseudothecia densities at the Cascade Head site, but site was also important for 3- and 4-year-old needles. Distance from coast is important to Swiss needle cast disease severity and needle retention in young stands, with Swiss needle cast impacts greater at lower elevation areas and closer to coast (Hansen et al., 2000; Rosso and Hansen, 2003; Lee et al., 2013; Shaw et al., 2014, 2021; Ritóková et al., 2021).

Disease severity and pseudothecial density measured on 2-year-old needles is highest in the upper crowns of plantation-grown Douglas-fir compared to middle and lower crown positions (Hansen et al., 2000; Manter et al., 2005; Shaw et al., 2014; Lan et al., 2019; Ritóková et al., 2021). Our pseudothecia density data across older needles is generally consistent with Douglas-fir crown vertical patterns for 2-year-old needles in plantations. The lower canopy position had consistently lowest density of pseudothecia for all needle age classes (Figure 6 and Table 3), while the upper canopy had the highest density, except the mid canopy of 4-year old needles in 2017.

Foliage diseases are generally thought to be most impactful in the highest humidity regions of a tree crown, which is typically the lower and inner crown (Tattar, 1989; Scharpf, 1993). In Christmas tree plantation settings in Pennsylvania and western Washington, *N. gaeumannii* was most severe in the lower and inner portion of the tree crown (Merrill and Longenecker, 1973; Chastagner and Byther, 1983). However, in western Oregon, disease severity of *N. gaeumannii* is consistently greatest in the upper crown for plantation trees (Hansen et al., 2000; Shaw et al., 2014; Ritóková et al., 2021), while the mid- and upper crown of mature trees show greater pseudothecia density in our study. This implies that environmental conditions for the fungus are more favorable in the mid and upper crown of mature trees, consistent with younger trees in western Oregon forests. Dye et al. (2020) analyzed the 22-year mean May through September low cloudiness (i.e., stratus, stratocumulus, and fog) and found a strong decline of low clouds in May and June, and a peak in July in the terrestrial highlands mode (Moose Mt. and Falls Creek). Seasonally, coastal fog frequency increases to a peak in August and is highest from June to September (Johnstone and Dawson, 2010). The coastal summer fog maintains needle wetness during the period of low summer precipitation and high summer temperatures. The coastal mode (Cascade Head, Klickitat Mt. and Woods Creek) had consistent low cloudiness that also peaked in late summer. The fog may wet the upper and mid crowns of co-dominant and dominant trees more than the lower canopy, and help explain canopy differences of *N. gaeumannii* density, although leaf wetness was not consistently higher on upper canopy foliage (Lan et al., 2019).

In western Oregon, winter temperature influences disease severity at a range of spatial and temporal scales (Manter et al., 2005; Stone et al., 2008a,b; Zhao et al., 2012; Lee et al., 2013, 2017; Wilhelmi et al., 2017). Leaf canopy temperature may be a key predictor of variations in *N. gaeumannii* density within the canopy of mature Douglas-fir. A wide range of biological responses from leaf photosynthesis (Jordan and Ogren, 1984) to net ecosystem exchange (Kim et al., 2016) are better predicted by leaf temperature than air temperature. Several thermal imaging and radiation absorption modeling studies show leaf temperature is higher in the upper crown than in the lower crown and understory (Still et al., 2019; Miller et al., 2021) due to more direct daytime solar irradiation at the treetop than at the bottom of the canopy (Sinoquet et al., 2001). The gradients in leaf canopy temperature are most likely an important factor for fungal development, notably in the cool winter months when temperatures are most limiting to the formation of pseudothecia. In coastal Oregon, pseudothecial primordia form in epistomatal chambers October to April following initial infection in the summer months (Stone et al., 2008a). Pseudothecia density was generally low (< 10%) for all needle age classes in mature trees, implying less colonization of the needles or slower growth within the needles. Winton et al. (2003) have demonstrated that pseudothecia density is significantly correlated with quantitative PCR, or the abundance of fungi within the needle. Therefore, other factors may be influencing colonization success of needles in mature trees. Although more recently, Montwé et al. (2021) contend that there is not a clear relationship between pseudothecia density, fungal DNA load and needle retention. They propose that there is a more complex unknown pathology involved in needle loss.

## CONCLUSION

*Nothophaeocryptopus gaeumannii* causes disease in plantation and forest trees when over 25% of the stomates of young (1 and 2-year-old) foliage are occluded, causing early needle casting. Our hypothesis that disease severity is lower in mature trees because pseudothecia emerge later on older needles is supported. Within the mature tree crowns, we also found that pseudothecia density varied with needle age, canopy position, and site. In older and mature trees, pseudothecia *of N. gaeumannii* were most abundant on 3- and 4-year-old needles and density of pseudothecia on all needles was very low (< 10%). Something about mature forest canopies and foliage appears to decrease the success of needle colonization or increase the time it takes for pseudothecia to emerge from the needles, in contrast to younger plantations (Lan et al., 2019), thus allowing the mature trees to have greater needle retention. Tree crown and canopy architectural differences may help explain these results, because mature trees have more complexities in microenvironmental patterns with deeper crowns, more shade, and less crown connectivity between trees, but this needs more studies in the future.

## Figures and Tables

**TABLE 1 |:** Summary of GLMM of pseudothecia incidence and density, across five study sites (15 trees) in western Oregon, tree age (mature or young trees), year, canopy positions and their interactions.

	Density					Incidence				
	Estimates	SE	z value	p value		Estimates	SE	z value	p value	
(Intercept)	−2.49	0.24	−10.56	<0.001	***	2.18	0.48	4.51	<0.001	***
treeageYoung	1.02	0.29	3.49	<0.001	***	2.22	0.95	2.33	0.020	*
canopyMiddle	−0.67	0.36	−1.86	0.063	.	1.87	0.64	2.91	0.004	**
canopyLower	−1.59	0.49	−3.20	0.001	**	0.00	0.37	0.00	1.000	
siteKlickitat Mt	−0.80	0.40	−2.00	0.046	*	−2.95	0.66	−4.50	<0.001	***
siteWoods Creek	−1.00	0.42	−2.37	0.018	*	−6.55	0.95	−6.89	<0.001	***
siteFalls Creek	−2.48	0.74	−3.37	0.001	***	−5.47	0.76	−7.22	<0.001	***
siteMoose Mt	−1.89	0.58	−3.28	0.001	**	−3.88	0.67	−5.81	<0.001	***
year2017	0.19	0.29	0.64	0.523		1.15	0.49	2.32	0.021	*
treeageYoung:canopyMiddle	0.25	0.43	0.58	0.559		−1.57	1.26	−1.25	0.213	
treeageYoung:canopyLower	0.34	0.57	0.60	0.550		0.29	1.15	0.25	0.800	
treeageYoung:siteKlickitat Mt	0.04	0.49	0.08	0.939		1.99	1.21	1.64	0.101	
treeageYoung:siteWoods Creek	0.87	0.49	1.76	0.078	.	4.01	1.34	2.99	0.003	**
treeageYoung:siteFalls Creek	1.50	0.79	1.90	0.057	.	4.90	1.31	3.75	<0.001	***
treeageYoung:siteMoose Mt	0.85	0.65	1.32	0.186		2.58	1.18	2.18	0.029	*
canopyMiddle:siteKlickitat Mt	−0.53	0.72	−0.73	0.465		−2.44	0.70	−3.49	<0.001	***
canopyLower:siteKlickitat Mt	−0.05	0.89	−0.06	0.952		−0.88	0.47	−1.87	0.061	
canopyMiddle:siteWoods Creek	−2.65	1.80	−1.47	0.141		−1.45	1.12	−1.29	0.196	
canopyLower:siteWoods Creek	1.62	0.69	2.37	0.018	*	1.29	0.89	1.45	0.148	
canopyMiddle:siteFalls Creek	0.36	1.12	0.32	0.749		−1.32	0.83	−1.59	0.113	
canopyLower:siteFalls Creek	1.85	1.04	1.77	0.077	.	1.27	0.61	2.07	0.038	*
canopyMiddle:siteMoose Mt	0.25	0.90	0.28	0.778		−1.35	0.71	−1.91	0.057	
canopyLower:siteMoose Mt	0.49	1.14	0.43	0.665		0.48	0.48	1.01	0.312	
treeageYoung:year2017	0.33	0.35	0.93	0.351		−0.85	1.20	−0.71	0.475	
canopyMiddle:year2017	0.25	0.48	0.53	0.599		−0.73	1.05	−0.70	0.485	
canopyLower:year2017	0.68	0.61	1.11	0.267		0.19	0.72	0.27	0.791	
siteKlickitat Mt:year2017	−0.45	0.61	−0.74	0.457		−1.92	0.62	−3.08	0.002	**
siteWoods Creek:year2017	−3.51	1.79	−1.96	0.050	*	−1.15	1.12	−1.02	0.308	
siteFalls Creek:year2017	−0.02	0.98	−0.02	0.986		−1.71	0.84	−2.02	0.044	*
siteMoose Mt:year2017	−0.57	0.86	−0.66	0.507		−2.92	0.70	−4.18	<0.001	***
treeageYoung:canopyMiddle:siteKlickitat Mt	0.43	0.82	0.52	0.601		1.89	1.42	1.33	0.183	
treeageYoung:canopyLower:siteKlickitat Mt	0.71	0.99	0.72	0.472		0.41	1.33	0.31	0.757	
treeageYoung:canopyMiddle:siteWoods Creek	3.54	1.83	1.94	0.053	.	3.76	1.73	2.17	0.030	*
treeageYoung:canopyLower:siteWoods Creek	−0.09	0.77	−0.12	0.907		1.31	1.65	0.80	0.425	
treeageYoung:canopyMiddle:siteFalls Creek	0.16	1.18	0.14	0.891		1.89	1.69	1.12	0.262	
treeageYoung:canopyLower:siteFalls Creek	−0.80	1.13	−0.71	0.478		−1.40	1.48	−0.95	0.345	
treeageYoung:canopyMiddle:siteMoose Mt	0.44	0.97	0.46	0.647		2.52	1.46	1.73	0.084	
treeageYoung:canopyLower:siteMoose Mt	0.84	1.21	0.70	0.486		1.11	1.42	0.78	0.434	
treeageYoung:canopyMiddle:year2017	−0.66	0.57	−1.16	0.246		0.44	1.91	0.23	0.818	
treeageYoung:canopyLower:year2017	−0.58	0.71	−0.82	0.414		−0.48	1.75	−0.28	0.782	
treeageYoung:siteKlickitat Mt:year2017	−1.37	0.75	−1.83	0.067	.	0.70	1.36	0.51	0.609	
treeageYoung:siteWoods Creek:year2017	1.74	1.83	0.95	0.340		−1.92	1.59	−1.21	0.227	
treeageYoung:siteFalls Creek:year2017	−0.37	1.04	−0.35	0.723		1.15	1.56	0.74	0.458	
treeageYoung:siteMoose Mt:year2017	−0.93	0.97	−0.96	0.338		0.09	1.35	0.07	0.947	
canopyMiddle:siteKlickitat Mt:year2017	−0.15	1.15	−0.13	0.899		1.97	1.16	1.70	0.089	
canopyLower:siteKlickitat Mt:year2017	−1.49	1.76	−0.85	0.397		−0.82	1.03	−0.80	0.421	
canopyMiddle:siteWoods Creek:year2017	−12.37	3909.49	0.00	0.997		0.73	1.67	0.44	0.662	
canopyLower:siteWoods Creek:year2017	−0.72	2.57	−0.28	0.781		−0.91	1.41	−0.65	0.517	
canopyMiddle:siteFalls Creek:year2017	−2.65	2.79	−0.95	0.341		0.65	1.37	0.48	0.632	
canopyLower:siteFalls Creek:year2017	−2.56	1.90	−1.34	0.179		−1.46	1.16	−1.26	0.209	
canopyMiddle:siteMoose Mt:year2017	1.08	1.20	0.90	0.371		−0.83	1.37	−0.61	0.544	
canopyLower:siteMoose Mt:year2017	0.31	1.50	0.21	0.835		−1.30	1.07	−1.22	0.224	
treeageYoung:canopyMiddle:siteKlickitat Mt:year2017	0.04	1.40	0.03	0.977		−2.04	2.09	−0.98	0.329	
treeageYoung:canopyLower:siteKlickitat Mt:year2017	1.74	1.90	0.92	0.360		0.30	2.02	0.15	0.880	
treeageYoung:canopyMiddle:siteWoods Creek:year2017	11.94	3909.49	0.00	0.998		−2.08	2.44	−0.85	0.393	
treeageYoung:canopyLower:siteWoods Creek:year2017	0.16	2.63	0.06	0.951		−0.65	2.30	−0.28	0.779	
treeageYoung:canopyMiddle:siteFalls Creek:year2017	2.52	2.84	0.89	0.374		−0.40	2.47	−0.16	0.873	
treeageYoung:canopyLower:siteFalls Creek:year2017	1.54	2.00	0.77	0.440		1.06	2.21	0.48	0.633	
treeageYoung:canopyMiddle:siteMoose Mt:year2017	−0.81	1.36	−0.60	0.550		0.07	2.22	0.03	0.975	
treeageYoung:canopyLower:siteMoose Mt:year2017	−0.47	1.64	−0.29	0.776		0.86	2.09	0.41	0.681	

Only 2-year-old needles were used.

Asterisks indicate significance at the 0.05 (*),

0.01 (**),

and 0.001 (***) levels.

Estimates are based on a logit transformation.

**TABLE 2 |:** Disease incidence (%) and pseudothecia density (%) of 2-year old needles of young plantations (data used to determine disease severity in Lan et al., 2019) and 2–6 year age classes on mature trees (*n* = 15).

Sites	Young plantation 2 year		Mature 2 year		Mature 3 year		Mature 4 year		Mature 5 year		Mature 6 year	
	2016	2017	2016	2017	2016	2017	2016	2017	2016	2017	2016	2017
**Incidence**												
Cascade Head	99.78 ± 0.22	100 ± 0	91.78 ± 2.32	97.78 ± 1.31	86.4 ± 9.27	93.11 ± 5.43	77.96 ± 3.79	90.01 ± 7.26	81.57 ± 7.97	97.22 ± 2.78	84.51 ± 1.35	85.71 ± 14.29
Klickitat Mountain	96.22 ± 1.61	85.78 ± 4.06	26.44 ± 7.39	10 ± 5.36	69.78 ± 7.03	41.12 ± 9.3	39.18 ± 6.96	50.19 ± 10.15	26.59 ± 12.55	34.65 ± 8.82	15.53 ± 7.99	28.04 ± 11.67
Woods Creek	94.89 ± 3.27	44.44 ± 8.07	2.22 ± 0.91	1.33 ± 0.58	30.22 ± 7.68	8.44 ± 2.84	33.78 ± 5.97	22.44 ± 4.71	8.12 ± 1.64	27.78 ± 4.2	9.33 ± 3.25	6.29 ± 2.29
Moose Mountain	96.89 ± 1.57	68.67 ± 9.88	22.44 ± 6.97	1.78 ± 0.91	54.89 ± 13.96	34.44 ± 10.94	52.51 ± 12.41	40 ± 12.41	51.59 ± 14.25	34.79 ± 11.06	39.5 ± 19.19	33.5 ± 12.82
Falls Creek	98.89 ± 0.59	97.33 ± 1.15	7.78 ± 2.3	2.44 ± 0.87	62.67 ± 10.39	38 ± 7.26	51.95 ± 6.97	65.79 ± 10.72	56.57 ± 8.21	51.66 ± 9.08	56.16 ± 12.14	53.64 ± 11.65
**Density**												
Cascade Head	12.82 ± 1.84	17.89 ± 3.08	4.56 ± 1.03	6.51 ± 1.34	7.52 ± 0.91	6.28 ± 0.7	6.63 ± 1.09	6.4 ± 0.88	7.36 ± 1.08	4.12 ± 0.57	9.33 ± 2.48	6.1 ± 0.7
Klickitat Mountain	6.22 ± 0.73	2.07 ± 0.38	1.84 ± 0.5	1.16 ± 0.52	2.04 ± 0.68	2.02 ± 0.47	2.68 ± 0.68	3.06 ± 1.18	2.36 ± 0.46	4.05 ± 1.96	1.42 ± 0.57	2.44 ± 1.42
Woods Creek	20.82 ± 2.07	4.65 ± 1.09	2.07 ± 1.14	0.07 ± 0.05	7.78 ± 2.41	1.59 ± 0.81	8.41 ± 1.78	6.94 ± 0.87	6.91 ± 1.6	5.7 ± 0.9	4.44 ± 1.24	2.72 ± 1.22
Moose Mountain	8.63 ± 1.18	3.18 ± 0.44	0.84 ± 0.15	1.25 ± 0.73	3.9 ± 0.7	4.51 ± 1.49	3.2 ± 0.73	8.82 ± 1.49	3.27 ± 1.07	6.63 ± 2.17	2.14 ± 0.51	4.07 ± 1.07
Falls Creek	7.93 ± 0.97	6.15 ± 1.75	0.71 ± 0.13	0.35 ± 0.27	8.55 ± 2.08	2.69 ± 0.58	5.54 ± 1.69	6.89 ± 1.36	3.98 ± 0.48	3.46 ± 1.05	3.95 ± 0.79	3.51 ± 0.89

**TABLE 3 |:** Summary of GLMM of pseudothecia density across five study sites (15 trees) in western Oregon, needle age (2–5-year-old), year, canopy position and their interactions.

	Estimate	SE	*z* value	*p* value	
(Intercept)	−2.32	0.26	−8.88	<0.001	***
canopyMiddle	−0.53	0.23	−2.25	0.024	*
canopyLower	−1.16	0.29	−4.07	<0.001	***
needleage3 year	−0.06	0.21	−0.31	0.759	
needleage4 year	−0.43	0.24	−1.78	0.075	.
needleage5 year	−0.20	0.28	−0.72	0.473	
siteKlickitat Mt	−0.99	0.42	−2.37	0.018	*
siteWoods Creek	−1.79	0.47	−3.80	<0.001	***
siteFalls Creek	−2.50	0.58	−4.33	<0.001	***
siteMoose Mt	−2.35	0.52	−4.48	<0.001	***
year2017	−0.14	0.06	−2.62	0.009	**
canopyMiddle:needleage3 year	0.45	0.32	1.41	0.159	
canopyLower:needleage3 year	0.69	0.37	1.84	0.065	.
canopyMiddle:needleage4 year	0.92	0.34	2.71	0.007	**
canopyLower:needleage4 year	1.14	0.39	2.92	0.004	**
canopyMiddle:needleage5 year	0.41	0.37	1.11	0.265	
canopyLower:needleage5 year	0.73	0.42	1.73	0.084	.
canopyMiddle:siteKlickitat Mt	−0.63	0.55	−1.14	0.253	
canopyLower:siteKlickitat Mt	−0.68	0.72	−0.95	0.341	
canopyMiddle:siteWoods Creek	−2.82	1.78	−1.59	0.113	
canopyLower:siteWoods Creek	1.20	0.54	2.21	0.027	*
canopyMiddle:siteFalls Creek	−0.47	0.93	−0.50	0.615	
canopyLower:siteFalls Creek	0.81	0.78	1.04	0.301	
canopyMiddle:siteMoose Mt	0.86	0.57	1.50	0.133	
canopyLower:siteMoose Mt	0.59	0.72	0.82	0.412	
needleage3 year:siteKlickitat Mt	0.18	0.40	0.44	0.663	
needleage4 year:siteKlickitat Mt	0.42	0.43	0.97	0.332	
needleage5 year:siteKlickitat Mt	−0.46	0.62	−0.73	0.465	
needleage3 year:siteWoods Creek	1.56	0.42	3.67	<0.001	***
needleage4 year:siteWoods Creek	2.49	0.43	5.79	<0.001	***
needleage5 year:siteWoods Creek	1.93	0.46	4.23	<0.001	***
needleage3 year:siteFalls Creek	2.67	0.53	5.02	<0.001	***
needleage4 year:siteFalls Creek	3.02	0.55	5.52	<0.001	***
needleage5 year:siteFalls Creek	2.03	0.59	3.46	<0.001	***
needleage3 year:siteMoose Mt	1.81	0.48	3.74	<0.001	***
needleage4 year:siteMoose Mt	2.61	0.50	5.22	<0.001	***
needleage5 year:siteMoose Mt	1.92	0.55	3.48	<0.001	***
canopyMiddle:needleage3 year:siteKlickitat Mt	−0.30	0.75	−0.40	0.692	
canopyLower:needleage3 year:siteKlickitat Mt	−0.13	0.91	−0.15	0.883	
canopyMiddle:needleage4 year:siteKlickitat Mt	−0.07	0.72	−0.10	0.918	
canopyLower:needleage4 year:siteKlickitat Mt	−0.09	0.89	−0.11	0.916	
canopyMiddle:needleage5 year:siteKlickitat Mt	1.12	0.85	1.32	0.186	
canopyLower:needleage5 year:siteKlickitat Mt	1.22	0.99	1.23	0.220	
canopyMiddle:needleage3 year:siteWoods Creek	2.63	1.81	1.45	0.147	
canopyLower:needleage3 year:siteWoods Creek	−1.76	0.67	−2.62	0.009	**
canopyMiddle:needleage4 year:siteWoods Creek	2.03	1.81	1.12	0.262	
canopyLower:needleage4 year:siteWoods Creek	−2.16	0.65	−3.31	<0.001	***
canopyMiddle:needleage5 year:siteWoods Creek	2.69	1.82	1.48	0.138	
canopyLower:needleage5 year:siteWoods Creek	−1.46	0.68	−2.15	0.031	*
canopyMiddle:needleage3 year:siteFalls Creek	−0.27	0.98	−0.27	0.785	
canopyLower:needleage3 year:siteFalls Creek	−1.55	0.86	−1.80	0.072	.
canopyMiddle:needleage4 year:siteFalls Creek	−0.49	0.99	−0.50	0.620	
canopyLower:needleage4 year:siteFalls Creek	−1.71	0.86	−1.98	0.048	*
canopyMiddle:needleage5 year:siteFalls Creek	0.40	1.02	0.40	0.693	
canopyLower:needleage5 year:siteFalls Creek	−0.82	0.90	−0.90	0.366	
canopyMiddle:needleage3 year:siteMoose Mt	−1.15	0.67	−1.72	0.086	.
canopyLower:needleage3 year:siteMoose Mt	−0.85	0.82	−1.04	0.299	
canopyMiddle:needleage4 year:siteMoose Mt	−1.51	0.67	−2.26	0.024	*
canopyLower:needleage4 year:siteMoose Mt	−1.21	0.81	−1.49	0.137	
canopyMiddle:needleage5 year:siteMoose Mt	−1.14	0.74	−1.54	0.123	
canopyLower:needleage5 year:siteMoose Mt	0.10	0.84	0.12	0.904	

Asterisks indicate significance at the 0.05 (*),

0.01 (**),

and 0.001 (***) levels.

Estimates are based on a logit transformation.

## Data Availability

The original contributions presented in the study are included in the article/supplementary material, further inquiries can be directed to the corresponding author.

## Footnotes

1 http://www.prism.oregonstate.edu/explorer/, accessed 11 April 2021.
